# Evaluation and Comparison of Multi-Satellite Aerosol Optical Depth Products over East Asia Ocean

**DOI:** 10.3390/toxics11100813

**Published:** 2023-09-26

**Authors:** Zhaoxiang Cao, Kuifeng Luan, Peng Zhou, Wei Shen, Zhenhua Wang, Weidong Zhu, Zhenge Qiu, Jie Wang

**Affiliations:** 1 College of Marine Sciences, Shanghai Ocean University, Shanghai 201306, China; 2 Estuarine and Oceanographic Mapping Engineering Research Center of Shanghai, Shanghai 200123, China; 3 School of Surveying and Land Information Engineering, Henan Polytechnic University, Jiaozuo 454000, China; 4 State Environmental Protection Key Laboratory of Satellite Remote Sensing, Aerospace Information Research Institute, Chinese Academy of Sciences, Beijing 100101, China; 5 College of Information Science, Shanghai Ocean University, Shanghai 201306, China

**Keywords:** aerosol optical depth, multi-source satellite, evaluation, East Asia Ocean

## Abstract

The atmosphere over the ocean is an important research field that involves multiple aspects such as climate change, atmospheric pollution, weather forecasting, and marine ecosystems. It is of great significance for global sustainable development. Satellites provide a wide range of measurements of marine aerosol optical properties and are very important to the study of aerosol characteristics over the ocean. In this study, aerosol optical depth (AOD) data from seventeen AERONET (Aerosol Robotic Network) stations were used as benchmark data to comprehensively evaluate the data accuracy of six aerosol optical thickness products from 2013 to 2020, including MODIS (Moderate-resolution Imaging Spectrometer), VIIRS (Visible Infrared Imaging Radiometer Suite), MISR (Multi-Angle Imaging Spectrometer), OMAERO (OMI/Aura Multi-wavelength algorithm), OMAERUV (OMI/Aura Near UV algorithm), and CALIPSO (Cloud-Aerosol Lidar and Infrared Pathfinder Satellite Observation) in the East Asian Ocean. In the East Asia Sea, VIIRS AOD products generally have a higher correlation coefficient (R), expected error within ratio (EE within), lower root mean square error (RMSE), and median bias (MB) than MODIS AOD products. The retrieval accuracy of AOD data from VIIRS is the highest in spring. MISR showed a higher EE than other products in the East Asian Ocean but also exhibited systematic underestimation. In most cases, the OMAERUV AOD product data are of better quality than OMAERO, and OMAERO overestimates AOD throughout the year. The CALIPSO AOD product showed an apparent underestimation of the AOD in different seasons (EE Below = 58.98%), but when the AOD range is small (0 < AOD < 0.1), the CALIPSO data accuracy is higher compared with other satellite products under small AOD range. In the South China Sea, VIIRS has higher data accuracy than MISR, while in the Bohai-Yellow Sea, East China Sea, Sea of Japan, and the western Pacific Ocean, MISR has the best data accuracy. MODIS and VIIRS show similar trends in R, EE within, MB, and RMSE under the influence of AOD, Angstrom exponent (AE), and precipitable water. The study on the temporal and spatial distribution of AOD in the East Asian Ocean shows that the annual variation of AOD is different in different sea areas, and the ocean in the coastal area is greatly affected by land-based pollution. In contrast, the AOD values in the offshore areas are lower, and the aerosol type is mainly clean marine type aerosol. These findings can help researchers in the East Asian Ocean choose the most accurate and reliable satellite AOD data product to better study atmospheric aerosols’ impact and trends.

## 1. Introduction

Aerosols are an essential component of the atmosphere that affect the atmosphere through two types of radiative forcing: direct radiative forcing, which changes the amount of solar radiation received at Earth’s surface through absorption and scattering, and indirect radiative forcing, which alters the radiative properties and lifetime of clouds by acting as cloud condensation nuclei [1,2,3,4]. The direct radiative forcing of aerosols is primarily influenced by aerosol optical depth (AOD), particle size distribution, and aerosol absorption properties [5,6,7]. For example, black carbon aerosols are the second most crucial atmospheric warming factor after carbon dioxide [8]. The uncertainty of black carbon content and its warming effect may exceed 200% compared to previously estimated warming effects [8,9]. In contrast, aerosols with weak absorption effects, for example, sulfate aerosols, have an important effect on the radiation balance of Earth’s surface by directly scattering solar radiation and indirectly changing the characteristics of cloud nodules [10,11]. The indirect radiative forcing of aerosols is mainly manifested as aerosols acting as cloud condensation nuclei, increasing the number of cloud droplets, thereby increasing the optical thickness and cloud albedo [12,13].

The AOD is one of the most important parameters for understanding the climate impact of aerosols, which can be calculated by integrating the aerosol extinction coefficient in the vertical direction [14,15]. The AOD can determine the size of the aerosol load, calculate the aerosol content, estimate PM 2.5, and reflect the degree and type of regional atmospheric pollution, so the study of AOD has important significance [16,17,18].

There are two main ways to obtain the AOD data: ground-based observations and satellite monitoring. Several global aerosol ground-based observation networks have been established worldwide for the study of global aerosols, such as the Aerosol Robotic Network (AERONET), the Chinese Sun-Spectral Observation Network (CSHNET), the Aerosol Network organized and implemented by the Chinese Academy of Sciences (SONET), and the Japanese Aerosol/Radiation Observation Network (SKYNET) [19,20,21,22]. Rahul et al. [23] studied the abnormally high AOD values in the Arctic region during the summer of 2011 using AERONET (Aerosol Robotic Network) and MICROTOPS II data, and the results showed that the high AOD was the result of fine-mode particles being transported to the Arctic by strong wind circulation. Although ground-based observation networks can provide real-time and accurate AOD values with errors of less than 0.01~0.02 [24], they can only reflect the AOD conditions in relatively small areas. To obtain long-term series and an extensive spatial range of the AOD, many satellites have been launched for global aerosol monitoring [16], such as the Moderate-resolution Imaging Spectroradiometer (MODIS), the Sea-Viewing Wide Field-of-View Sensor (SeaWiFS), TROPOMI’s successor sensor Ozone Monitoring Instrument (OMI), the Multi-angle Imaging Spectro Radiometer (MISR), the Cloud-Aerosol Lidar and Infrared Pathfinder Satellite Observation (CALIPSO), etc. [16,25,26,27]. These sensors play an essential role in aerosol monitoring and development. Larisa et al. [28] analyzed the changes in AOD in China over the past 20 years using ATSR (Along-Track Scanning Radiometer) and MODIS data. They found that ATSR and MODIS showed similar seasonal AOD trends. The maximum AOD value shifted from the south in spring to the east coast in the summer. Luan et al. [29] used CALIPSO satellite data to find that, with the implementation of pollution prevention and control policies in northern China and the Yangtze River Delta region, the AOD values have decreased, and the values of the aerosol extinction coefficient at different altitudes in the vertical direction have also significantly decreased. However, with time, satellite aging is inevitable. Some satellite data services have been discontinued [30]. Still, at the same time, excellent new-generation sensors for atmospheric monitoring have been developed, such as the Visible Infrared Imaging Radiometer Suite (VIIRS). Retrieval algorithms have also been developed from the early contrast reduction method to the current Lidar Technology [31], Multi-Angle Implementation of Atmospheric Correction (MAIAC) algorithm [18], and other algorithms; for example, OMI provides two groups of standard aerosol products by different algorithms, OMAERO and OMAERUV [16,32]. These have all promoted the development of new sensors and the updating of satellite AOD products. Scientists have done much work to verify the AOD products generated by new AOD satellite data and new inversion algorithms. Liu et al. [33] validated the AOD monitoring results of MODIS (MYD04 3K) and CALIPSO in China based on AERONET data. They found that the spatiotemporal distribution patterns of the two-satellite data were the same. However, particularly in regions with high AOD values in China, the CALIPSO data system was lower than that of MODIS. Su et al. [34] evaluated and compared the AOD products of MODIS Deep Blue (DB), MODIS Dark Target (DT), MODIS DT/DB Merge, and VIIRS in eastern China at regional and site scales from 2016 to 2020. The results showed that in eastern China, the accuracy of the Deep Blue algorithm on Terra/Aqua MODIS and NPP VIIRS platforms was better than that of the Dark Target algorithm.

Therefore, we can see that satellite AOD products have been extensively evaluated globally and regionally in recent years [27,35,36,37]. However, there has been little satellite data validation around the East Asian Sea, and it is not known which AOD retrieval algorithm can achieve higher accuracy in the East Asian Sea. In the ocean, due to the limited number of ground observation stations, the retrieval of AOD using single satellite data is not representative and may lead to weak credibility. Therefore, the use of multiple satellites and different monitoring instruments for AOD research can alleviate this problem [38]. This integrated analysis method can improve the reliability and accuracy of the AOD data, especially in remote sea areas [39]. Many scholars have validated satellite AOD data on different land surfaces. Gupta et al. [40] validated the global land surface Terra MODIS data set from 2000 to 2015 and the Aqua MODIS data set from 2003 to 2015 through AERONET data, and the results showed that Terra MODIS showed more changes over time. Ajtai et al. [41] validated aerosol optical depth (AOD) from Spinning Enhanced Visible Infrared Radiometer (SEVIRI) data using data from six AERONET stations in Romania and the Baltic. Due to the limited availability of measured data over the ocean, in this study, we referred to the previous experience [42,43]. For example, Gassó et al. [42] used AERONET to assess the accuracy of OMI 388 nm data over the global ocean and found that OMAERUV does an adequate job of suppressing cloudy scenarios within instrument capabilities and that OMAERUV bias remains within limits for higher aerosol optical depths. Ettehadi et al. [43] verified the aerosol optical depth of MODIS Dark Target 10 KM, MODIS MAIAC 1 KM, and VIIRS product (AERDB_L2) over the Black Sea using AERONET data. The results showed that the correlation coefficient between satellite AOD retrieval and AERONET AOD measurement was between 0.45 and 0.93, and the root mean square error (RMSE) was between 0.047 and 0.159, indicating the reliability of satellite AOD retrieval in this area. We selected AERONET sites in the East Asian region to accurately validate satellite data. The sites we used were near coastlines, islands, and offshore oil drilling platforms, making it reasonable to show the AOD situation over the sea [43].

This study aims to comprehensively validate satellite-derived AOD products in the East Asian seas and explore potential factors affecting the accuracy of these products. In this work, AERONET data were used as a reference to evaluate the overall performance of MODIS, VIIRS, MISR, OMAERO, OMAERUV, and CALIPSO AOD products in the region, and the accuracy of satellite data was assessed at regional and individual AERONET sites during different seasons. Additionally, the impacts of AOD, Angstrom exponent (AE), and precipitation on the AOD retrievals were investigated. The overall evaluation of satellite data, the AOD frequency distributions at different spatial scales, and seasonal assessments are described in Section 3.1 and Section 3.2, respectively. Section 3.3 presents the spatiotemporal distribution characteristics of the AOD in the East Asian seas. The impacts of AOD, AE, and precipitable water on the AOD retrievals are discussed in Section 4, followed by a summary of the study’s main findings in Section 5.

## 2. Materials and Methods

### 2.1. Satellite Data

MODIS is a remote sensing instrument that comes from the Terra and Aqua satellites launched by the National Aeronautics and Space Administration (NASA) of the United States in 1999 and 2002, respectively. Terra and Aqua satellites are sun-synchronous polar orbiting satellites with an orbit altitude of 705 km. MODIS provides observations in 36 spectral bands and completes global words every 1–2 days with a scanning swath of 2330 km [44]. The MODIS data used in this article is from the ‘AOD_550_Dark_Target_Deep_Blue_Combined_Mean’ dataset of the MOD08_D3 product of the Terra satellite. The AOD product is a combination of DT and DB AOD datasets, and the combination process relies on the normalized difference vegetation index [45]. The AOD product provides greater spatial coverage on land, especially in transition areas [25]. The DT algorithm is mainly used to retrieve AOD in visually “dark” areas (such as vegetation-covered ground). Kaufman et al. [46] found that in many vegetation-covered areas, the surface reflectance is consistent at 0.47 m, 0.65 m, and 2.11 µm. The DB algorithm was originally designed for bright reflective surfaces that the DT algorithm does not support, such as deserts. One of the main principles of the DB algorithm is that the reflectivity of most surfaces is very low at 0.412 µm, which increases the relative contribution of aerosol to the observed TOA reflectivity [24]. The DB algorithm is an effective alternative solution in areas where the DT algorithm does not work [47]. Additionally, the AOD data are filtered from the 10-km resolution level-2 daily product with a quality assurance (QA) value of 3 [48].

The Suomi NPP satellite is part of the NOAA Joint Polar Satellite System (JPSS). It was jointly established by NASA and the National Oceanic and Atmospheric Administration (NOAA) of the United States. It provides essential weather observation data for NOAA’s National Weather Service and contributes to NASA’s Earth climate research. VIIRS is a scanning radiometer with a swath width of 3040 km and 22 bands with a wavelength range of 412 nm–1250 nm. It can monitor cloud and aerosol properties, ocean color, ocean and land temperature, ice movement, temperature, fires, and Earth reflectivity. VIIRS was designed to inherit the MODIS sensor and is optimized in terms of instrument performance, pixel selection, and band settings. This means that VIIRS will not follow MODIS in monitoring aerosol optical thickness. The zenith angle range of VIIRS is 70°, which is larger than the 64° of MODIS. Therefore, the accuracy of VIIRS AOD products tends to be more sensitive to surface and aerosol property assumptions [49]. Currently, VIIRS only offers DB aerosol products. The VIIRS AOD used in this study is the ‘Aerosol_Optical_Thickness_550_Land_Ocean_Mean’ dataset in the AERDB_D3 data [49,50].

MISR is carried on the Terra satellite and was launched in 1999. MISR operates on an orbit 705 km above sea level, using nine cameras to collect radiation in four bands (446, 558, 672, and 866 nm) at fixed angles. Different angle radiation observations provide a method for distinguishing different types of atmospheric particles, cloud formation, and surface coverage [51]. However, in the MISR retrieval algorithm, no regional/seasonal constraints are imposed on the aerosol type, and the surface bi-directional reflectance factor is estimated by empirical orthogonal functions (EOFs), assuming only that the normalized angular shapes are spectrally similar [52]. MISR is essential for identifying non-spherical aerosols, but its spectral range is limited, so it is not sensitive to the presence of sub-visible cirrus [53].

OMI is a sensor carried on the NASA Aura Earth Observing System satellite, launched on 15 July 2004, and is part of the A-train satellite formation. OMI began collecting data on 9 August 2004. OMI has a swath width of 2600 km composed of 60 pixels on the ground, with each pixel size of 13 × 24 km^2^, and can monitor atmospheric pollutants such as global aerosols, O_3_, SO_2_, NO_2_, HCHO, etc. The AOD inversion products of OMI include OMAERO [27] and OMAERUV [27,54,55]. OMAERO is based on the multi-wavelength algorithm, using up to 20 bands between 331 nm and 500 nm, and selects the best aerosol value from the L2 high-quality data reported from each grid [27,56]. OMAERUV is an aerosol retrieval algorithm based on two near-ultraviolet wavelengths, utilizing spectral information at 354 and 388 nm wavelengths [57].

The CALIPSO satellite was jointly developed by the National Aeronautics and Space Administration (NASA) and the French National Space Research Center (CNES). It was launched in June 2006 and operates in a sun-synchronous orbit with an orbit inclination of 98.2° and a height of 705 km. CALIOP (Cloud-Aerosol Lidar with Orthogonal Polarization) is a dual-wavelength polarized laser radar, the first continuous observation spaceborne lidar, and an effective tool for detecting cloud and atmospheric aerosol features. The aerosol retrieval algorithm of CALIPSO is mainly divided into three steps: identifying the hierarchical characteristics in the 532 nm attenuation backscatter signal, distinguishing the hierarchical types by using the scene classification method and the three-channel data, and finally obtaining the particle extinction characteristics by using the hybrid extinction retrieval algorithm [58]. It provides much research data for studying global climate and environmental changes. In the experiment, we found that CALIPSO L2 data have few matches with AERONET, and L3 data have higher data screening criteria than L2 data. So, we chose the CALIPSO Level 3 Tropospheric Aerosol Profile product CAL_LID_L3_tropospheric_APro_CloudFree-Standard-V4 and selected the parameter of aerosol optical thickness. The time resolution of the CALIPSO Level 3 data product is one month, the spatial resolution is 2° × 5°, the vertical resolution is 60 m, and the detection height range is from sea level to 12.1 km, with a total of 208 layers. Due to the limited number of matches between CALIPSO instantaneous data and AERONET sites, as a polar satellite, CALIPSO has long observation intervals and large orbit intervals and cannot obtain continuous observation data of the same region. This may lead to the omission of short-term and small-scale pollution activities. At the same time, the Level 3 long-term monthly data product, to some extent, compensates for this drawback and is more reasonable when studying the regional scope. In addition, CALIPSO monthly data have many matches with AERONET data, so we chose CALIPSO monthly data as the data evaluation product [2,59,60,61]. We also provide the occurrence frequency (OF) of dusty marine and clean marine aerosol types for each AERONET site in 2013–2020. CALIPSO can retrieve the aerosol type using information such as the laser-detected declination ratio, backscatter coefficient, subsurface type, and aerosol height. These data are derived from the CALIPSO L3 aerosol-type data and have been extensively used with reasonable data accuracy [62].

For data comparability, we interpolated the CALIPSO spatial resolutions to 1° × 1° [63]. The six-satellite data in this paper are used from 2013 to 2020. Table 1 gives basic information about the satellite data products used in this paper.

### 2.2. AERONET Data

**Figure 1:**
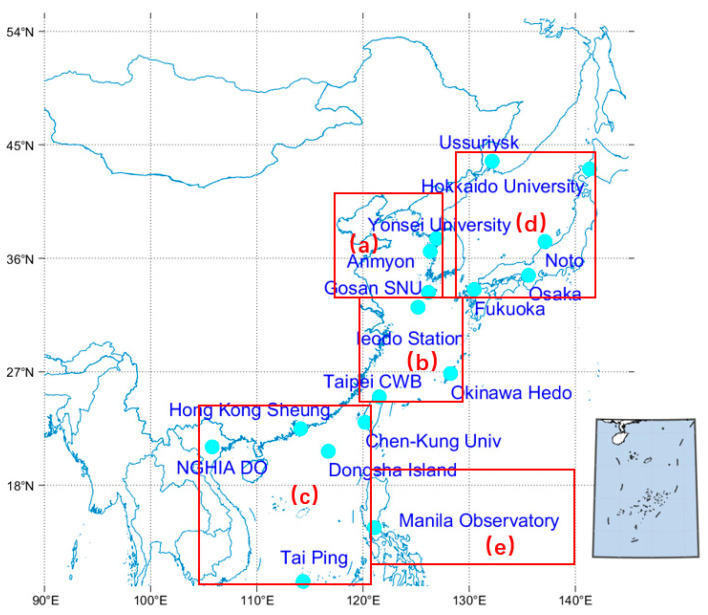
Distribution of AERONET stations used in East Asian seas. (**a**): Bohai-Yellow Sea; (**b**): East China Sea; (**c**): South China Sea; (**d**): Japan Sea; (**e**): Western Pacific Sea.

AERONET is a global aerosol observation network established by NASA and other institutions [20]. It provides optical, microphysical, and radiative data on aerosols, with the central monitoring equipment being the CE-318 sun photometer manufactured by CIMEL [64]. AERONET provides effective AOD bands at 340 nm, 380 nm, 440 nm, 500 nm, 675 nm, 870 nm, and 1020 nm. The AOD data used in this study are Level 2.0 products that have been quality-assured and filtered for clouds. Seventeen AERONET sites with long-term observation periods in East Asia were selected to validate the accuracy of satellite AOD retrieval based on the research time and range [16]. All the selected sites are located near the coasts of East Asian cities or surrounded by oceans, as shown in Figure 1. Table 2 gives basic information about the AERONET site used in this article. We also divided the East Asian seas into several parts for study, namely the Bohai-Yellow Sea (32–41° N, 119–126° E), the East China Sea (23–32° N, longitude 117–131° E), the South China Sea (10–23° N, 105–118° E), the Sea of Japan (37–43° N, 131–139° E), and the western part of the Pacific Sea (120–140° N, 5–18° E). The OF of dusty and clean marine aerosols in each AERONET station from 2013 to 2020 is shown in Table 2, and they are calculated from Table 2. The OF of aerosol types is calculated by the percentage of dusty and clean marine aerosols in the horizontal grid unit and the height layer of the seven aerosol samples [62].

### 2.3. Method

The matching scheme between the satellite and AERONET is to average the AERONET data within a time window of 30 min of the satellite overpass; then, if there are multiple AERONET observations in the grid unit of the satellite, the spatial average is carried out [16,65].

To maintain consistency and comparability, the 440 nm, 500 nm, and 675 nm bands of AERONET, the 342.5 nm, 442 nm, and 483.5 nm bands of OMAERO, and the 354 nm, 388 nm, and 500 nm bands of OMAERUV were interpolated using a second-order polynomial interpolation method to obtain the AOD values at the 550 nm wavelength [16]. The difference in wavelength between the 558 nm band of MISR and the 532 nm and 550 nm bands of CALIPSO is small, so this difference was ignored [66].

(1) $$\ln\tau_{\partial }=\partial_{0}+\partial_{1}\ln\lambda +\partial_{2}{\left(\lambda \right)}^{2}$$

where $\tau_{\alpha }$ represents the AOD value at the wavelength λ. $\partial_{I}$(*I* = 0, 1, 2) are unknown coefficients [67,68,69], it can be estimated by the least square method in combination with AOD values of other known bands of AERONET.

The study uses Pearson’s correlation coefficient (R), root-mean-square error (RMSE), median bias (MB), and expected error (EE) as indicators to verify the accuracy of satellite-retrieved AOD. When the satellite-retrieved AOD value falls within the expected error (EE within), the satellite retrieval is considered good. The calculation methods for RMSE, MB, and EE are as follows:

(2) $$\mathrm{RMSE}=\sqrt{\frac{1}{n}\overset{n}{\underset{i=1}{\sum }}(\tau_{\mathrm{satellite}}-\tau_{\mathrm{aeronet}}{)}^{2}}$$

(3) $$\mathrm{MB}=\frac{1}{n}\overset{n}{\underset{i=1}{\sum }}(\tau_{\mathrm{satellite}}-\tau_{\mathrm{aeronet}})$$

(4) $$\mathrm{EE}=\pm (0.05+0.15*\tau_{\mathrm{aeronet}})$$

where $\tau_{aeronet}$ represents the AOD value from AERONET, $\tau_{satellite}$ represents the AOD value from the satellite, and n represents the number of satellite–AERONET data pairs with temporal and spatial matching [16,70]. It should be noted that the range of EE varies with factors such as region, time-lapse, AERONET site location, and season [45,71]. In the East Asia region studied by us, the coastline sites and some island sites have relatively serious pollution due to the anthropogenic impact of land-based sources, i.e., higher AOD values. It can also be seen from Table 2 that the AERONET sites in the East Asian Sea generally have a high OF of dusty marine aerosols. To better analyze EE within and compare different satellite data, we unified the EE calculation method for all stations [72].

To avoid the influence of abnormal satellite data on the accuracy evaluation, this study excluded data pairs in the accuracy evaluation where the difference between satellite and the AERONET AOD values was more significant than three times the AERONET standard deviation.

## 3. Results

### 3.1. Overall Assessment of Satellite Data Products

**Figure 2:**
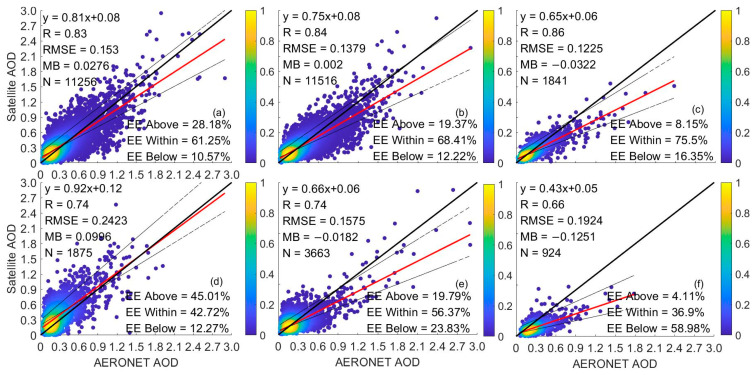
Scatter density plots of satellite data matched with AERONET data. (**a**): MODIS; (**b**): VIIRS; (**c**): MISR; (**d**): OMAERO; (**e**): OMAERUV; (**f**): CALIPSO.

Figure 2 shows the scatter plot of the AOD from various satellites and the AERONET AOD in the East China Sea from January 2013 to December 2020. We can see some differences in the accuracy of the AOD data from different sensors in the East China Sea. The number of matches between MODIS and VIIRS AOD products and the AERONET AOD is close and more significant than other satellite data. The difference in precision between MODIS and VIIRS AOD data is small. Although the slope of the fitting line between MODIS and AERONET AOD data is slightly higher than that of the VIIRS AOD products, VIIRS has a higher correlation coefficient, lower RMSE and MB, and a higher EE within ratio than MODIS. MISR has the highest R and EE within compared to other satellite products, which is consistent with the results of Chen et al. [48], but due to its narrow swath width, its matching number is only 1/6~1/7 of the MODIS/VIIRS matching number [32,48]. The precision of OMI’s two algorithm products is relatively lower than that of MODIS, VIIRS, and MISR, and OMAERO’s intercept is the largest (0.09), which indicates that OMAERO’s monitoring deviation is serious when the AOD is low (0 < AOD < 0.1). However, the slope of OMAERO closest to 1 suggests that its aerosol model setting may be more suitable for the study area [47]. The OMAERUV AOD product has a higher EE within ratio than OMAERO and lower RMSE and MB than OMAERO, but its correlation coefficient R is lower than that of OMAERO. Generally, three main factors affect the inversion results of OMI AOD: (1) interference from clouds; (2) incorrect estimation of surface reflectance; and (3) incorrect assumptions about aerosol profiles [73,74]. Sub-pixel cloud pollution in OMI pixels is one of the biggest sources of uncertainty in AOD retrieval in OMI measurement [56,74]. From the scatter plot of CALIPSO data, it can be seen that CALIPSO underestimates AERONET data to a greater extent; CALIPSO’s main reason for 532 nm AOD underestimation is the failure of detecting the entire aerosol layer due to signal attenuation, as indicated by Kacenelenbogen et al. (2011) [75], and Torres et al. (2013) [76]. This is similar to the accuracy evaluation results of CALIPSO in most of the East Asia region [33].

**Figure 3:**
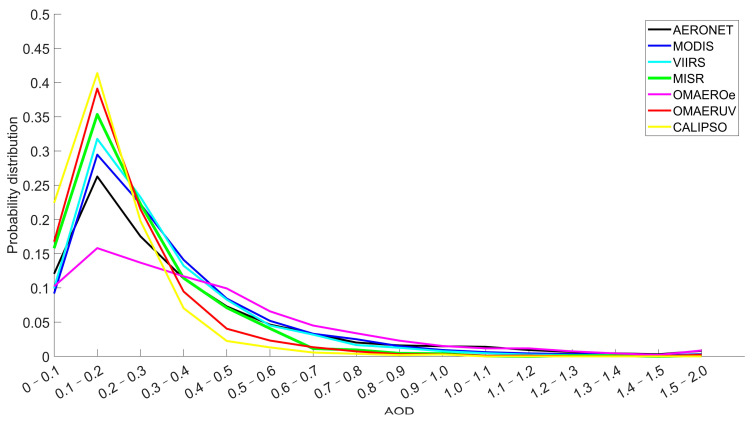
Satellite inversion and AERONET monitoring of the AOD matching histograms.

Figure 3 shows the logarithmic line plots of the satellite AOD matched with AERONET AOD. The horizontal axis represents the AOD value, and the vertical axis represents the percentage of corresponding the satellite AOD to the total matched pairs between the satellite and AERONET [26]. All the satellite data show similar distribution patterns to AERONET, with peaks appearing at 0.1–0.2, which confirms that the AOD over the East China Sea is concentrated at low values [77]. When the AOD is between 0 and 0.1, the probability distributions of OMAERO, MODIS, and VIIRS are lower than those of AERONET. With the increase in the AOD range, when the AOD is 0.1–0.2, only OMAERO is lower than AERONET, which indicates that the OMAERO value may be more distributed in the part greater than 0.2. When the AOD was less than 0.2, the frequency difference between CALIPSO data and AERONET data was the largest, indicating that the AOD monitoring sampling of CALIPSO mainly occurred in the smaller AOD range. Meanwhile, when the AOD is greater than 0.3, the OMAERUV always has a lower frequency than the AERONET.

**Figure 4:**
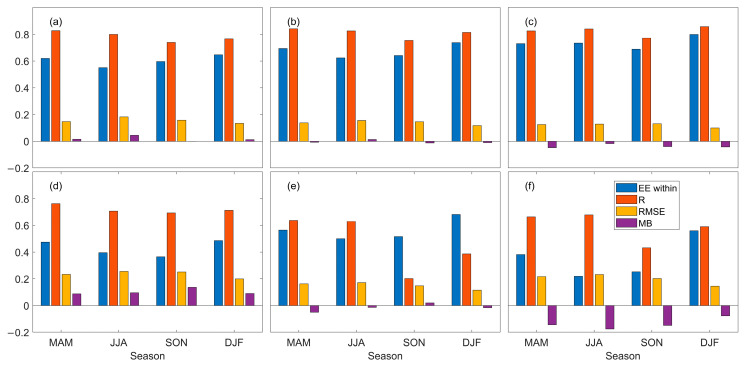
Seasonal EE within, R, RMSE, and MB histograms calculated from satellite products matched with the AERONET AOD data. (**a**): MODIS; (**b**): VIIRS; (**c**): MISR; (**d**): OMAERO; (**e**): OMAERUV; (**f**): CALIPSO.

Figure 4 displays the seasonal variation of satellite data and AERONET AOD, providing a better understanding of the accuracy of satellite data in different seasons over the East China Sea. As shown in the figure, the seasonal trends in the statistical data of MODIS and VIIRS AOD are generally consistent, with VIIRS exhibiting higher data accuracy in spring and summer than MODIS. The deviation in MODIS data may be related to errors caused by seasonal clouds and unreasonable aerosol model assumptions [78,79]. The inversion effect of MODIS data is better in autumn and winter than in spring and summer, consistent with the results of Mao et al. (2020) using MODIS data in the East Asian region [80]. MISR has a relatively high EE within the year, with MB less than 0 in all seasons, indicating an overall underestimation of the AOD, consistent with its response in most areas of the world. The underestimation may be due to MISR’s field of view angle and cloud effects [32,48,81]. Of the two algorithm products of OMI, OMAERO has a higher RMSE, with MB greater than 0 throughout the year and the lowest data accuracy in autumn. Although the accuracy of the OMAERUV data is also unstable, the deviation from AERONET is smaller than that of OMAERO. The accuracy of the CALIPSO data varies significantly in different seasons, with significant underestimation of the AOD, especially in summer, which may be due to the increase in aerosol load caused by biomass burning and dust transmission in summer, as well as the large amount of water vapor in the air affecting the aerosol size. The CALIPSO AOD obtained from the lidar ratio strongly depends on aerosol size, which is also closely related to aerosol type. Therefore, the significant deviation is caused by the incorrect choice of lidar ratio in the CALIPSO AOD inversion algorithm [33].

### 3.2. Regional Scale Evaluation

After presenting the overall accuracy of VIIRS in the East Asian Seas, the inconsistent AOD sampling times and different station locations in the East Asian Seas may lead to satellite statistics that do not represent the situation well. Table A1 and Table A2 provide the R, RMSE, MB, and EE within different satellite AOD products compared to AERONET in other sea areas to better understand the precision performance of satellite data at each site. Overall, the accuracy of satellite data does not show a trend with latitude or longitude. The AOD data accuracy of MISR is stable and superior to other sensors, which, to some degree, demonstrates the superiority of the multi-angle imaging algorithm in ocean areas. This may be because the multi-angle algorithm does not require a priori information on surface properties, making it suitable for all types of surfaces [82]. However, MISR and CALIPSO show a certain degree of negative bias in different sea areas, indicating that they both underestimate the AOD values. In the Bohai Sea, East China Sea, and Sea of Japan regions, the R of MODIS, VIIRS, and MISR products are all above 0.8, EE within are all above 59%, and there are low RMSE and MB. In the South China Sea, the MODIS, VIIRS, and MISR data perform very similarly, but at this time, the MODIS AOD product data accuracy is superior to MISR. Meanwhile, although OMAERUV has lower R and EE in the South China Sea than OMAERO and CALIPSO, it has RMSE and MB closer to 0. In the western Pacific Ocean, although OMAERO has a higher R than MODIS and VIIRS, its EE within is lower, and RMSE and MB are more elevated, indicating unstable data accuracy.

**Figure 5:**
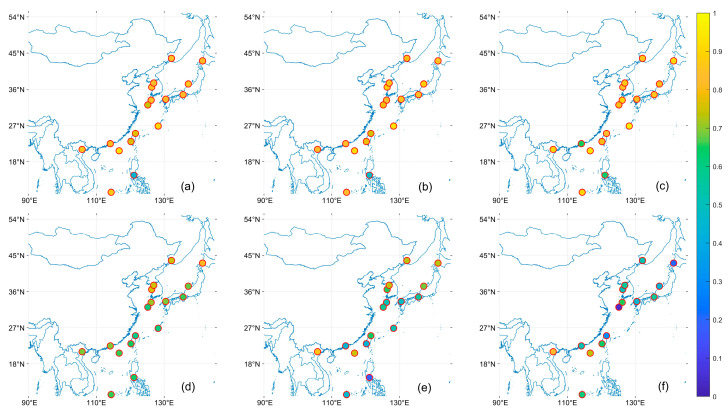
Distribution of R for satellite data products based on the AERONET AOD comparison. (**a**): MODIS; (**b**): VIIRS; (**c**): MISR; (**d**): OMAERO; (**e**): OMAERUV; (**f**): CALIPSO.

**Figure 6:**
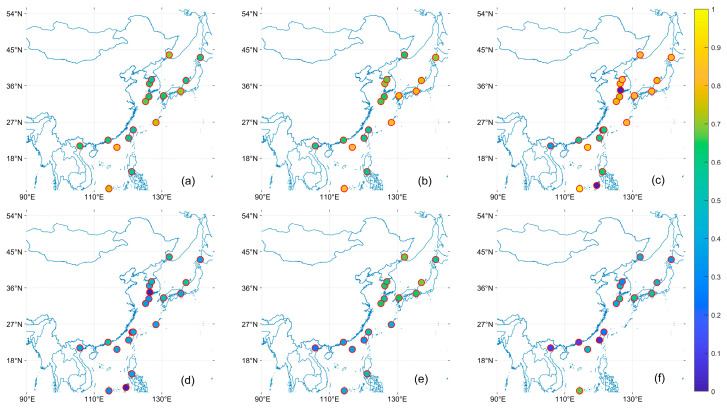
Distribution of EE within for satellite data products based on the AERONET AOD comparison. (**a**): MODIS; (**b**): VIIRS; (**c**): MISR; (**d**): OMAERO; (**e**): OMAERUV; (**f**): CALIPSO.

**Figure 7:**
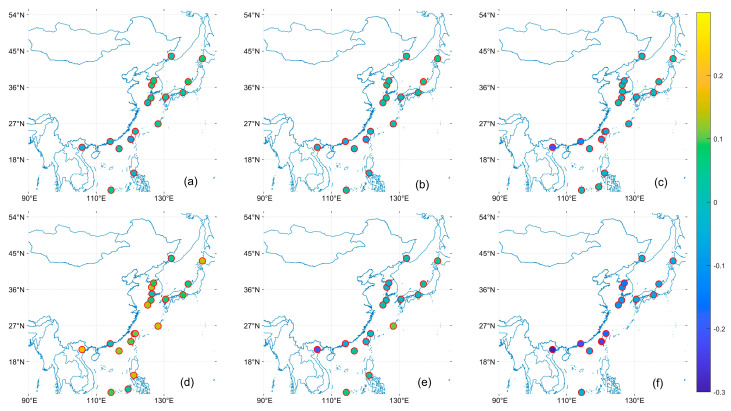
Distribution of MB for satellite data products based on the AERONET AOD comparison. (**a**): MODIS; (**b**): VIIRS; (**c**): MISR; (**d**): OMAERO; (**e**): OMAERUV; (**f**): CALIPSO.

Figure 5, Figure 6 and Figure 7 shows the R, EE within, and MB of the satellite AOD product with respect to the AERONET site. In the East China Sea region, sites with low R are mainly located in the East China Sea area, which is more evident under the OMAERO, OMAERUV, and CALIPSO satellites. In the Bohai Sea and Japan Sea regions, MISR has higher EE within than MODIS and VIIRS and closer to 0 MB, indicating higher accuracy of MISR data in these areas. However, MISR performs poorly in NGHIA DO, the Pearl River Delta, and Taiwan, which may be due to the high AOD values in these regions, consistent with the conclusion of Chen et al. that MISR has significant errors under high AOD [48]. All products performed poorly in the Philippines region near the western Pacific, with significantly lower R and EE within than other regions and higher MB. In general, the study can find that the distribution of EE within tends to be larger the farther away from the land. This may be related to the parameter setting of our EE, such as Tai Ping, Okinawa Hedo, and Dongsha Island sites, which are less affected by human activities, so the AOD value is small, resulting in a larger EE within [25].

### 3.3. Spatial and Temporal Distribution Characteristics of the AOD in East Asian Seas

**Figure 8:**
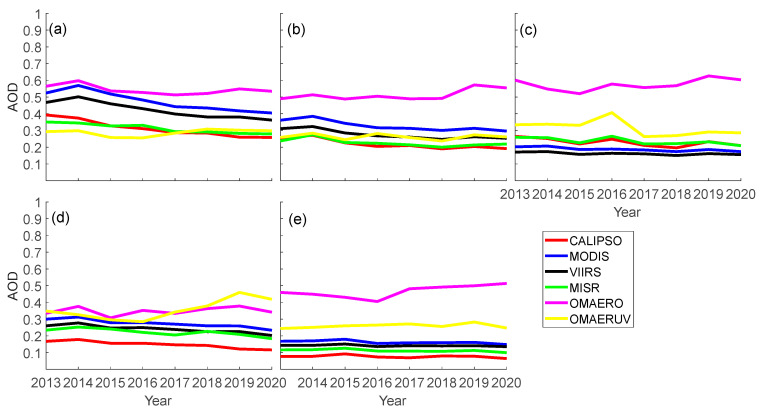
The year-by-year AOD averages of satellite data in the East Asian seas from 2013 to 2020. (**a**): Bohai-Yellow Sea; (**b**): East China Sea; (**c**): South China Sea; (**d**): Japan Sea; (**e**): Western Pacific Sea.

Figure 8 shows the annual average changes of the AOD in the Bohai-Yellow Sea, East China Sea, South China Sea, Japan Sea, and the western Pacific Ocean from 2013 to 2020. Firstly, it can be observed that the AOD values in each sea area are relatively small, and the inter-annual AOD changes from 2013 to 2020 are also relatively small, indicating that the AOD values are relatively stable. Overall, the annual average AOD value in the Bohai Sea region has significantly decreased over the years, which may be related to the improved air quality in the North China region and the reduced pollution transported from land to the ocean [29,83]. At the same time, the OMAERO sensor generally shows higher annual AOD in the East Asian seas than other sensors, which is consistent with the results of Livingston et al. in Mexico and Europe [56,84]. The difference between the annual AOD values obtained by OMAERO and those obtained by other sensors is also relatively significant. This may indicate that OMAERO cannot reasonably represent the AOD in the East Asian seas compared to MODIS, VIIRS, MISR, OMAERUV, and CALIPSO sensors. Interestingly, due to the sampling period effect, although the data performance at monthly/daily resolution is generally average, the annual AOD values shown by CALIPSO have a better agreement with other sensors over a long time series, especially in the Bohai-Yellow Sea and East China Sea regions. OMAERUV exhibits significantly different AOD values from other sensors in the Japan Sea and the western Pacific Ocean. The annual AOD values in these regions are relatively low, which may be related to the standard transportation of human-induced pollution from the mainland [59]. In the South China Sea and the western Pacific Ocean, the AOD values are generally low. 

**Figure 9:**
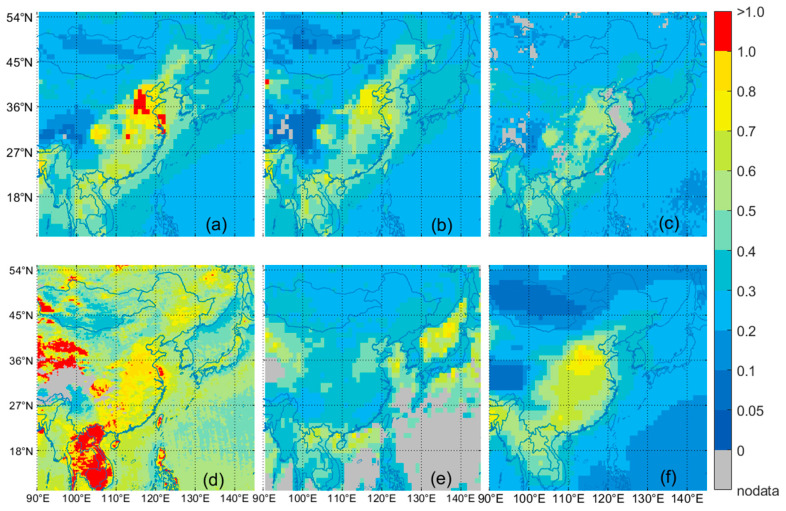
Spatial distribution of the mean AOD values in the East Asian seas from 2013 to 2020. (**a**) MODIS (550 nm); (**b**) VIIRS (550 nm); (**c**) MISR (558 nm); (**d**) OMAERO (550 nm); (**e**) OMAERUV (550 nm); (**f**) CALIPSO (532 nm).

**Figure 10:**
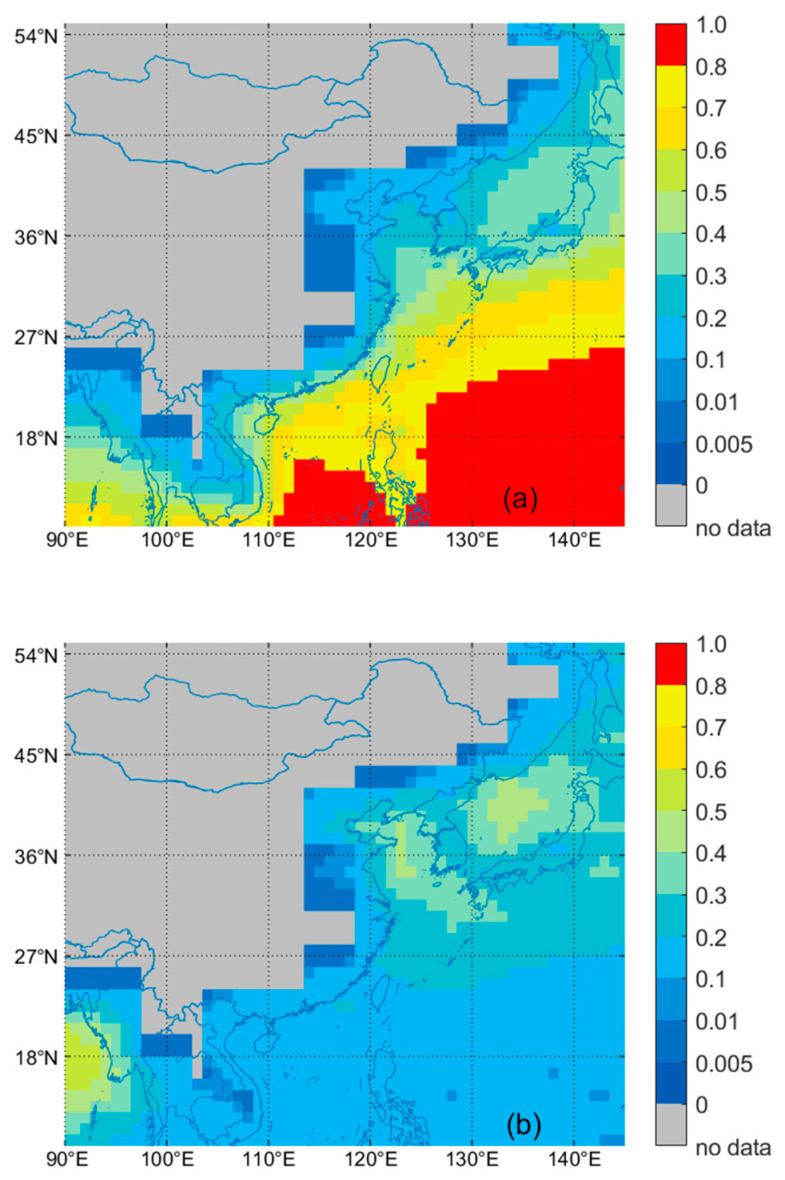
Spatial distribution of clean marine aerosol and dusty marine aerosol OF based on CALIPSO data for 2013–2020. (**a**): clean marine aerosol OF; (**b**): dusty marine aerosol OF.

The spatial distribution of annual AOD in the East Asian seas from 2013 to 2020 based on MODIS, VIIRS, MISR, OMAERO, OMAERUV, and CALIPSO is shown in Figure 9. In the calculation of AOD seasonal mean distribution, only the grid points with the proportion of observed values greater than 5% during the study period were selected to participate in the calculation of spatial distribution. Figure 9 shows that OMAERO has a higher AOD value than other satellite sensors, which may be because OMI has no reasonable surface albedo library and the influence of pixel size in cloud detection [56,85]. Comparative analysis reveals significant spatial distribution characteristics of the AOD in the East Asian seas. The spatial distribution features of MODIS, VIIRS, MISR, and CALIPSO are more similar, with high values mainly appearing in the Bohai-Yellow Sea and coastal areas. According to research by Shikwambana et al. [25,86,87,88,89], the high AOD values in marine areas are mainly caused by three factors: (1) the AOD in coastal areas is influenced by aerosols from the mainland; (2) aerosols are transported across the ocean, and continental aerosols can affect remote ocean areas; and (3) sea spray forms sulfate aerosols, which is the primary mechanism of marine aerosol formation. Therefore, we can infer that the high AOD values in coastal areas may be due to the influence of aerosols from the mainland. The frequency distribution of marine-type aerosols in Figure 10 shows that the Bohai-Yellow Sea and the Sea of Japan are mainly affected by dust-type marine aerosols, indicating significant land-source influence, particularly the impact of East Asian dust transport, leading to the high AOD values in these areas [29,90,91]. The AOD values in the western Pacific seas are low, mainly due to clean marine-type aerosols, mainly sea salt aerosols formed by the rupture of bubbles [92]. However, it cannot be ruled out that the discharge of pollutants from ships may also contribute to the aerosol source [93]. OMAERUV shows high AOD values in the Sea of Japan, possibly due to the significant impact of the near-ultraviolet algorithm in the inversion of the AOD over thin convective clouds and clear sky cover over the ocean [73].

## 4. Discussion

Based on the analysis above, it can be seen that different satellite data have different levels of uncertainty in the East Asian Sea area. The differences in satellite retrieval of the AOD are related to factors such as sampling period, algorithm, aerosol model, and atmospheric environmental conditions [47]. This section will discuss the satellite retrieval performance and error reasons under different scenarios based on three factors: AOD, AE, and precipitation water.

### 4.1. Effect of AOD Magnitude on Satellite Inversion of the AOD

Table 3, Table 4 and Table 5 shows the performance of MODIS, VIIRS, MISR, OMAERO, OMAERUV, and CALIPSO at different AOD ranges. Figure 2 indicates that the most frequent AOD range is 0–0.5, and the overall AOD situation may not fully reflect the accuracy of satellite data. To better understand the accuracy of data in different the AOD ranges, we divide the 0–0.5 range into five parts: 0–0.1, 0.1–0.2, 0.2–0.3, 0.3–0.4, and 0.4–0.5, and seek the differences in satellite accuracy in different the AOD ranges (see Table 3, Table 4 and Table 5). We find significant differences in data accuracy characteristics in different AOD ranges. In the 0–0.5 range, the VIIRS AOD product is superior to MODIS data in the 0–0.4 range, indicating that the latest DB algorithm updated surface model has improved the accuracy of the AOD retrieval [16,94,95]. In the 0–0.5 range, the CALIPSO product underestimates the AOD values, and the data accuracy gradually deteriorates with the increase in the AOD. However, when the AOD is in the range of 0–0.1, CALIPSO’s MB is closest to 0 compared to other sensors, and data performance is second only to MISR and better than other sensors. When the AOD is in the range of 0.1–0.2 and 0.2–0.3, MISR performs the best, with MB less than 0.01, EE within a ratio higher than 0.75, and RMSE of 0.0528 and 0.0790, respectively. This may be related to MISR’s multi-angle imaging algorithm being more sensitive to aerosol absorption [3]. When the AOD is 0.3–0.4, VIIRS exhibited higher R and EE within than MISR, while OMAERO still shows poor data accuracy. The two OMI algorithm products have large deviations when the AOD is 0–0.1; the real reason is that at OMI’s coarse spatial resolution, low AODs are always affected by cloud contamination effects [96,97,98].

### 4.2. Effects of AE and Precipitation Water on Satellite Inversion of the AOD

**Figure 11:**
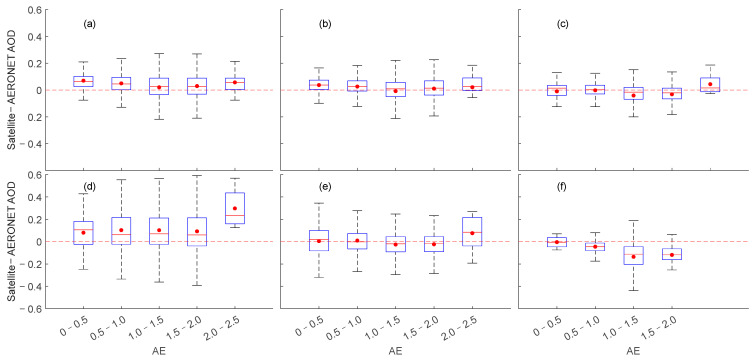
Box plots of the AOD errors (satellite—AERONET) relative to AERONET AE for different satellite data over the East Asian Sea. Where (**a**): MODIS; (**b**): VIIRS; (**c**): MISR; (**d**): OMAERO; (**e**): OMAERUV; (**f**): CALIPSO.

Figure 11 shows the absolute retrieve deviation (i.e., the difference between satellite retrieval and ground observation) of the AOD from satellite data within different AE ranges. AE is used as a qualitative indicator of aerosol particle size, with values between 0 and 2, with values below 1 indicating large particles such as sea salt and dust, and values above 1 indicating the m parameter range of small particles in the atmosphere associated with urban industrial aerosols or biomass combustion [99]. We can find that the absolute inversion deviation of different satellite data varies with the size of AE. MODIS and VIIRS exhibit the same pattern of change but with some differences in data accuracy. Overall, VIIRS has a lower absolute deviation, while VIIRS maintains lower error in the higher AE range (2.0 < AE < 2.5). MISR shows a lower deviation when AE < 1.0. At the same time, it exhibits apparent underestimation when 1.0 < AE < 2.0, which may be because the main components of aerosols within this range are mixed-mode and fine-mode particles, while the MISR algorithm only includes two non-spherical parts of the mid-mode and coarse-mode aerosol simulations, resulting in data deviation of MISR when 1.0 < AE < 2.0 [52,100]. OMAERO exhibits higher positive absolute error, while the near-UV algorithm product OMAERUV shows stable deviation change. When AE is in the range of 0–2.0, the mean and median fluctuate around 0. OMAERUV AOD Level 3 product uses the latest carbonaceous aerosol model to improve the identification of carbonaceous and desert dust aerosols by using carbon monoxide columns (CO) measured by the AIRS sensor on the Aqua satellite, as well as new climatology based on aerosol layer height from CALIPSO data. This may be the reason why the impact of AE on OMAERUV inversion of the AOD is relatively small in the East Asian Sea [73,96,101]. CALIPSO data show minimal absolute error in the AOD when 0 < AE < 0.5, with high data quality. However, when AE > 0.5, the negative fundamental error is obvious, which may be related to the underestimation of the AOD in CALIPSO data.

**Figure 12:**
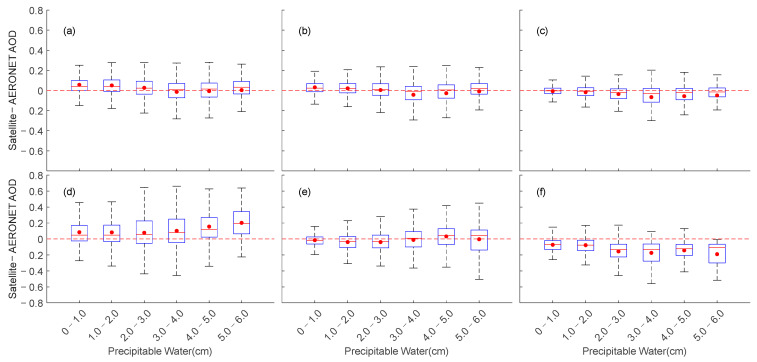
Box plots of the AOD errors (satellite-AERONET) relative to AERONET PW for different satellite data over the East Asian Sea. Where (**a**): MODIS; (**b**): VIIRS; (**c**): MISR; (**d**): OMAERO; (**e**): OMAERUV; (**f**): CALIPSO.

Precipitable water is believed to be closely related to the hygroscopic growth of aerosols [93], and the absorption and diffusion of aerosols may be the main reasons for the uncertainty of global and regional simulated precipitable water changes [102]. Whether different precipitable water significantly impacts the accuracy of satellite data near AERONET sites in the East China Sea is also an important consideration. Figure 12 shows the absolute inversion deviation of the AOD from satellite data within different precipitable water ranges. When the precipitable water range is 0–1 cm, the MISR and AERONET AOD observations are highly consistent, with small AOD deviation and maximum and minimum deviation values close to 0. However, when precipitable water is greater than 1 cm, MISR shows a negative AOD absolute inversion deviation. The VIIRS and MODIS products are similarly affected by precipitable water. When precipitable water is between 0–1.0 cm and 1.0–2.0 cm, the AOD deviation is generally greater than 0 for MODIS, VIIRS, and OMAERO inversion of the AOD results, while VIIRS has lower AOD variation than MODIS and OMAERO between 0–3.0 cm. However, VIIRS has an AOD deviation closer to 0 when precipitable water is between 2 and 3 cm, while MODIS has a smaller AOD deviation between 3 and 4 cm. The MISR and VIIRS products perform better than the other four AOD products when precipitable water is relatively high (5 cm < precipitable water < 6 cm). The OMAERUV AOD products also have a low AOD deviation when precipitable water is between 0 and 0.1 cm, but the data accuracy fluctuates greatly with increasing precipitable water.

## 5. Conclusions

Based on AERONET data as the reference, the performance of MODIS, VIIRS, MISR, OMAERO, OMAERUV, and the CALIPSO AOD products in the East Asian waters from 2013 to 2020 was evaluated. All six satellite data products correlated well with AERONET, with correlations higher than 0.65. MISR had the highest EE within and R and the lowest RMSE, indicating the stability of the multi-angle algorithm in the East Asian Sea. Still, its number of matchups was significantly lower than that of MODIS and VIIRS. VIIRS had a minor MB, reaching −0.0041, and its data accuracy was better than MODIS, but MODIS and VIIRS AOD showed similar seasonal variation trends in statistical data. OMAERO had the closest fit line slope to 1 with AERONET, indicating that its aerosol model setting might be more reasonable, but the overall data accuracy was lower than that of OMAERUV. Due to sampling time and lidar signal-to-noise ratio settings, CALIPSO data underestimated the AOD throughout the year. Regarding regional satellite data performance, MISR, MODIS, and VIIRS showed stable and similar data accuracy in each sea area, with MISR performing relatively better. Still, the MODIS data accuracy was better than that of MISR in the South China Sea. In the western Pacific waters, the performance of all products was significantly poorer, which may be due to the lack of sufficient AERONET stations in the region to select appropriate aerosol models.

The interannual variation of the AOD in the Bohai-Yellow Sea is closely related to the pollution from the adjacent land. The AOD values in the ocean areas close to the ground are higher, mainly due to the diffusion of pollutants. In contrast, the aerosols in the atmosphere over the open ocean, far from the land, show lower AOD values.

The impact of AOD on satellite retrievals shows significant differences in satellite data accuracy for different AOD ranges. For the AOD values in the range of 0–0.1, the CALIPSO product performs best. The MISR data have the best performance for the AOD values in the fields of 0.1–0.2 and 0.2–0.3, with a small MB of less than 0.01, an EE within ratio higher than 0.75, and RMSE values of 0.0528 and 0.0790, respectively. For the AOD values in the range of 0.3–0.4, the VIIRS data accuracy is slightly better than MISR. As the successor sensor of MODIS, the VIIRS data trend changes similarly to MODIS when affected by AE but with higher data accuracy. For precipitable water, values of 2–3 cm, VIIRS data accuracy is better than MODIS, while the opposite is true for precipitable water values of 3–4 cm. The OMAERO AOD data have a deviation more significant than 0 for different precipitable water and AE ranges, while the CALIPSO AOD product is smaller than 0. The OMAERUV AOD product is more stable than OMAERO and CALIPSO under the influence of precipitable water and AE.

This article also has some limitations. For example, for MODIS, VIIRS, OMI, and MISR, we consider daily data, but for lidar data, it is more reasonable to use monthly data for validation due to the availability of data, especially too few data points for data matching, which may affect the accuracy comparison to some extent.

In summary, for the overall AOD analysis of the East Asian Sea area, we recommend using VIIRS L3 data, which have high retrieval accuracy and reliable confidence. If the focus is on studying the most suitable algorithm and its improvement in the East Asian Sea area, we recommend using MISR data, whose multi-angle algorithm has significant advantages in this region. When the AOD is low (0 < AOD < 0.1), we recommend using CALIPSO data. CALIPSO data perform well for long-time series studies and can provide high-quality information on aerosol type and vertical distribution. The OMI near-ultraviolet algorithm product OMAERUV performs moderately, while the multi-wavelength algorithm product OMAERO still requires improvements in aerosol models and algorithms to reduce errors.

## Figures and Tables

**Table 1:** Basic information about the satellite data products.

Character of Sensor	Sensor-Satellite	Product Name	Product Algorithm	The Band Used in This Paper (nm)	Spatial Resolution
Spectral instrument	MODIS	MOD08_D3	Dark_Target_Deep_Blue_Combined	550	1° × 1°
	VIIRS	AERDB_D3	Deep Blue	550	1° × 1°
	OMAERO	OMI-Aura_L3-OMAEROe	Multi-Wavelength method	342.5, 442, 483.5	0.25° × 0.25°
	OMAERUV	OMI-Aura_L3-OMAERUVd	Near-Ultraviolet	354, 388, 500	1° × 1°
Multi-view angular sensor	MISR	MIL3DAEN_4	Multi-view angle	558	0.5° × 0.5°
Active lidar sensor	CALIPSO	CAL_LID_L3_Tropospheric_APro_CloudFree	Lidar	532	2° × 5°

**Table 2:** AERONET site information with marine-type aerosols at the site location. The OF of dusty and clean marine aerosols is calculated from CALIPSO L3 data, averaged from 2013 to 2020.

Site Name	Longitude	Latitude	Elevation (m)	AOD Effective Monitoring Period	Dusty Marine OF	Clean Marine OF
Ieodo Station	125.182	32.123	29	2013–2019	0.23	0.30
Chen-Kung Univ	120.205	22.993	50	2002–2020	0.08	0.46
Dongsha Island	116.729	20.699	5	2003–2020	0.06	0.53
Gosan SNU	126.161	33.292	72	2001–2016	0.24	0.29
Okinawa Hedo	128.249	26.867	60	2019–2020	0.12	0.54
Anmyon	126.33	36.54	47	1999–2020	0.22	0.11
Fukuoka	130.47	33.52	30	2012–2020	0.15	0.19
Hong Kong Sheung	114.12	22.483	40	2012–2018	0.08	0.36
Manila Observatory	121.08	14.64	63	2009–2020	0.03	0.58
NGHIA DO	105.80	21.05	40	2010–2019	0.04	0.10
Noto	137.14	37.33	200	2001–2020	0.14	0.18
Osaka	135.59	34.65	50	2000–2020	0.16	0.21
Tai Ping	114.36	10.38	4	2012–2020	0.03	0.81
Taipei CWB	121.54	25.01	26	2000–2020	0.12	0.39
Ussuriysk	132.16	43.70	280	2004–2019	0.02	0.01
Yonsei University	126.93	37.56	97	2011–2020	0.18	0.08
Hokkaido University	141.34	43.08	59	2015–2020	0.18	0.22

**Table 3:** Comparison of MB differences between satellite products and AERONET AOD matching data in different AOD ranges.

	MB					
AOD Range	MODIS	VIIRS	MISR	OMAERO	OMAERUV	CALIPSO
0–0.1	0.0542	0.0449	0.0166	0.1026	0.0681	−0.0037
0.1–0.2	0.0539	0.0358	0.0060	0.1137	0.0267	−0.0443
0.2–0.3	0.0455	0.0226	−0.0076	0.0994	−0.0266	−0.0792
0.3–0.4	0.0277	0.0049	−0.0367	0.1114	−0.0777	−0.1321
0.4–0.5	0.0134	−0.0189	−0.0784	0.0978	−0.1397	−0.2178

**Table 4:** The representation is the same as shown in Table 3. But for the EE within.

	EE within					
AOD Range	MODIS	VIIRS	MISR	OMAERO	OMAERUV	CALIPSO
0–0.1	0.6387	0.7212	0.8857	0.4221	0.5982	0.8261
0.1–0.2	0.6567	0.7554	0.8810	0.4303	0.6988	0.5469
0.2–0.3	0.6232	0.7058	0.7874	0.4011	0.5550	0.4322
0.3–0.4	0.6174	0.6907	0.6498	0.4691	0.3902	0.2586
0.4–0.5	0.5600	0.6026	0.6160	0.4274	0.2872	0.1341

**Table 5:** The representation is the same as shown in Table 4. but for the RMSE.

	RMSE					
AOD Range	MODIS	VIIRS	MISR	OMAERO	OMAERUV	CALIPSO
0–0.1	0.0870	0.0688	0.0405	0.1702	0.1217	0.0560
0.1–0.2	0.1024	0.0773	0.0528	0.2081	0.1063	0.0765
0.2–0.3	0.1236	0.1011	0.0790	0.2242	0.1123	0.1164
0.3–0.4	0.1370	0.1145	0.1108	0.2418	0.1537	0.1759
0.4–0.5	0.1738	0.1499	0.1300	0.2561	0.2003	0.2409

## Data Availability

MODIS, VIIRS (https://ladsweb.modaps.eosdis.nasa.gov, accessed on 10 January 2023), OMI (https://disc.gsfc.nasa.gov, accessed on 15 February 2023), MISR (https://misr.jpl.nasa.gov, accessed on 15 February 2023), AERONET (https://aeronet.gsfc.nasa.gov, accessed on 5 January 2023). CALIPSO (https://www-calipso.larc.nasa.gov, accessed on 5 February 2023).

## Appendix A

**Table A1:** From satellite data R and EE within the Bohai-Yellow Sea, East China Sea, South China Sea, Sea of Japan, and the western Pacific Ocean.

Region	AERONET	R/EE within					
		MODIS	VIIRS	MISR	OMAERO	OMAERUV	CALIPSO
Bohai-Yellow Sea	Yousei Anmyon Gosan	0.84/0.59	0.86/0.67	0.87/0.79	0.74/0.41	0.63/0.58	0.57/0.43
East China Sea	Ieodo Taipei CWB Okinawa	0.81/0.64	0.83/0.67	0.86/0.72	0.60/0.37	0.57/0.50	0.12/0.22
South China Sea	Dongsha_Island	0.84/0.66	0.84/0.68	0.84/0.67	0.67/0.40	0.59/0.34	0.67/0.28
	Tai Ping						
	Hong Kong						
	NGHIA DO						
	Chen-Kung						
Sea of Japan	Noto	0.83/0.64	0.85/0.73	0.85/0.81	0.72/0.46	0.64/0.63	0.44/0.46
	Osaka						
	Fukuoka						
	Ussuriysk						
	Hokkaido						
Western Pacific Ocean	Manila	0.45/0.64	0.47/0.73	0.63/0.81	0.63/0.46	0.18/0.63	/

**Table A2:** The representation is the same as shown in Table 3. but for the RMSE and MB.

		RMSE/MB					
Region	AERONET	MODIS	VIIRS	MISR	OMAERO	OMAERUV	CALIPSO
Bohai-Yellow Sea	Yousei Anmyon Gosan	0.17/0.06	0.14/0.03	0.11/−0.02	0.23/0.10	0.14/−0.03	0.16/−0.12
East China Sea	Ieodo Taipei CWB Okinawa	0.15/0.01	0.13/−0.001	0.11/−0.02	0.23/0.13	0.20/0.03	0.14/−0.09
South China Sea	Dongsha_Island	0.16/−0.01	0.16/−0.03	0.17/−0.09	0.28/0.10	0.23/−0.04	0.25/−0.20
	Tai Ping						
	Hong Kong						
	NGHIA DO						
	Chen-Kung						
Sea of Japan	Noto	0.11/0.05	0.09/0.03	0.08/−0.01	0.17/0.08	0.10/0.01	0.10/−0.06
	Osaka						
	Fukuoka						
	Ussuriysk						
	Hokkaido						
Western Pacific Ocean	Manila	0.13/−0.02	0.14/−0.03	0.11/−0.03	0.26/0.15	0.16/0.03	/
